# 3-(4-Hydroxy-3-methoxyphenyl) propionic acid contributes to improved hepatic lipid metabolism via GPR41

**DOI:** 10.1038/s41598-023-48525-3

**Published:** 2023-12-01

**Authors:** Ryuji Ohue-Kitano, Yuki Masujima, Shota Nishikawa, Masayo Iwasa, Yosuke Nishitani, Hideaki Kawakami, Hiroshige Kuwahara, Ikuo Kimura

**Affiliations:** 1 Laboratory of Molecular Neurobiology, Graduate School of Biostudies, Kyoto University, Yoshidakonoe-cho, Sakyo-ku, Kyoto, 606-8501 Japan; 2 Laboratory of Molecular Endocrinology, Graduate School of Pharmaceutical Sciences, Kyoto University, Sakyo-ku, Kyoto, 606-8501 Japan; 3 Center for Living Systems Information Science (CeLiSIS), Kyoto University, Sakyo-ku, Kyoto, 606-8501 Japan; 4 Research Center, Maruzen Pharmaceuticals Co., Ltd., Fukuyama, Hiroshima 729-3102 Japan; 5 Department of Applied Biological Science, Graduate School of Agriculture, Tokyo University of Agriculture and Technology, Fuchu, Tokyo 183-8509 Japan

**Keywords:** Fat metabolism, Nutrient signalling, Endocrine system and metabolic diseases

## Abstract

3-(4-hydroxy-3-methoxyphenyl) propionic acid (HMPA) is a metabolite produced by the gut microbiota through the conversion of 4-hydroxy-3-methoxycinnamic acid (HMCA), which is a widely distributed hydroxycinnamic acid-derived metabolite found abundantly in plants. Several beneficial effects of HMPA have been suggested, such as antidiabetic properties, anticancer activities, and cognitive function improvement, in animal models and human studies. However, the intricate molecular mechanisms underlying the bioaccessibility and bioavailability profile following HMPA intake and the substantial modulation of metabolic homeostasis by HMPA require further elucidation. In this study, we effectively identified and characterized HMPA-specific GPR41 receptor, with greater affinity than HMCA. The activation of this receptor plays a crucial role in the anti-obesity effects and improvement of hepatic steatosis by stimulating the lipid catabolism pathway. For the improvement of metabolic disorders, our results provide insights into the development of functional foods, including HMPA, and preventive pharmaceuticals targeting GPR41.

## Introduction

Phenolic compounds, which are highly prevalent phytochemicals, are found in edible plants, fruits, and beverages in our daily life^1^. In recent decades, the health benefit of polyphenols has been extensively explored, focusing on their antioxidant, anti-inflammatory, antibacterial, antiadipogenic, and neuroprotective properties in both animal models and humans^2,3^. Conversely, several studies have revealed that a significant portion of dietary polyphenols remains unabsorbed in the small intestine; as a result, they accumulated in the colon where they undergo extensive metabolism by the gut microbiota^4^. The gut microbiota play a crucial role in transforming and metabolizing the original polyphenolic structures into smaller metabolites that can be easily absorbed and contribute to various beneficial effects on the host^5^. To gain insight into the physiological functions of dietary polyphenols and their metabolites, comprehensive investigations on their bioaccessibility and bioavailability dynamics are necessary. However, the mechanisms underlying the interplay among dietary polyphenols, gut microbiota, and host health remain poorly understood.

3-(4-Hydroxy-3-methoxyphenyl) propionic acid (HMPA), classified as a hydroxycinnamic acid, is a phenolic acid that represents the reduced form of 4-hydroxy-3-methoxycinnamic acid (HMCA). HMPA is a natural polyphenolic compound found in various bacteria and plants. Recently, Santamaría et al.^6^ unveiled that *L*. *plantarum* (plant-origin bacteria), harbors hydroxycinnamate reductase, an enzyme that acts as a heterodimeric NADH-dependent coumarate reductase, facilitating the conversion of HMCA to HMPA. Furthermore, HMPA may be transformed in the intestine through the metabolic activity of gut bacteria from phenolic acids and various flavonoids^7,8^. Among phenolic acids, hydroxycinnamic acids such as HMCA and caffeic acid are prominently found in foods and beverages, particularly in coffee, which is one of the most extensively, consumed beverages worldwide. Notably, the ingestion of coffee, appreciated for its considerable concentration of hydroxycinnamic acids, results in the presence of detectable levels of HMPA in the bloodstream^9^. Conversely, a study conducted on individuals who had ileostomy without a colon demonstrates reduced detectability of HMPA following the consumption of coffee predominantly rich in hydroxycinnamic acid esters (caffeoylquinic acids and chlorogenic acid) compared with normal coffee^10^, supporting that its production occurs in the colon. Indeed, we have also reported that HMCA is metabolized by the gut microbiota, belonging to various microbes of the phylum Bacteroidetes, into HMPA in mice^11^. Thus, HMPA is a polyphenolic compound that can abundantly exist in our body through dietary intake or metabolism by gut bacteria. Therefore, precise comprehension of its absorption and distribution dynamics in the body is crucial to understanding the physiological regulatory effects of HMPA.

In addition to some of the other phenolic metabolites, HMPA exhibits beneficial health effects such as antidiabetic properties^12^, anticancer activities^13^, and cognitive function improvement^14^. Moreover, HMPA exhibits anti-obesity effects and improves hepatic lipid metabolism in high-fat diet (HFD)-induced obesity mice^11^. Furthermore, several mechanisms underlying the beneficial physiological effects of polyphenolic compounds have been elucidated. Several host receptors, including the aryl hydrocarbon receptor (AhR), toll-like receptor (TLR) 4, zeta-chain-associated protein kinase (ZAP)-70, and 67-kDa laminin receptor (67LR), have been identified as receptors for various dietary polyphenols and their microbial metabolites^15–18^. AhR was reported to bind to numerous polyphenolic molecules including gut metabolites such as gallic acid and urolithins and exhibit inhibitory effects on inflammatory bowel disease and anticancer activity^15^. Likewise, TLR4 contributes toward improvement in the cognitive function by curcumin in experimental models of brain injury^16^ and has chemoprevention effects against carcinogen-induced skin tumorigenesis by resveratrol^16^. 67LR and ZAP-70 have been characterized as epigallocatechin gallate (EGCG) receptors, mediating anticancer and anti-allergic effects^17,18^. A recent in silico study screened polyphenol-binding proteins targeting 50 polyphenols, suggesting the involvement of several receptors in polyphenol interactions^19^. In an in silico study, Stefaniu et al.^19^ indicated the potential affinity of HMCA toward G protein-coupled receptors (GPCRs). Among GPCRs, GPR41 and GPR43 have been identified as receptors for short-chain fatty acids (SCFAs), which are representative metabolites of the gut microbiota and reported to contribute to anti-obesity effects and hepatic metabolic improvements^20–23^. Nevertheless, the affinity of HMPA toward the host receptor has not been evaluated until now, and the molecular mechanisms underlying the modulation of the anti-obesity effects and the improvement of HMPA-mediated hepatic lipid metabolism have not been thoroughly clarified.

Therefore, this study aimed to explore the beneficial metabolic effects of HMPA on a mouse model of HFD-induced obesity and the molecular mechanism underlying these effects. In addition, a global analysis of the changes in liver gene expression following HMPA administration was performed to elucidate the detailed mechanisms of the improvement in liver lipid metabolism. Furthermore, an exploratory study was conducted to identify specific receptors for HMPA in the body. Finally, whether the gut bacteria-produced HMPA following the intake of HMCA exerts beneficial metabolic effects via the host receptors were examined.

## Results

### Abrogation of HFD-induced obesity by HMPA intake

**Figure 1:**
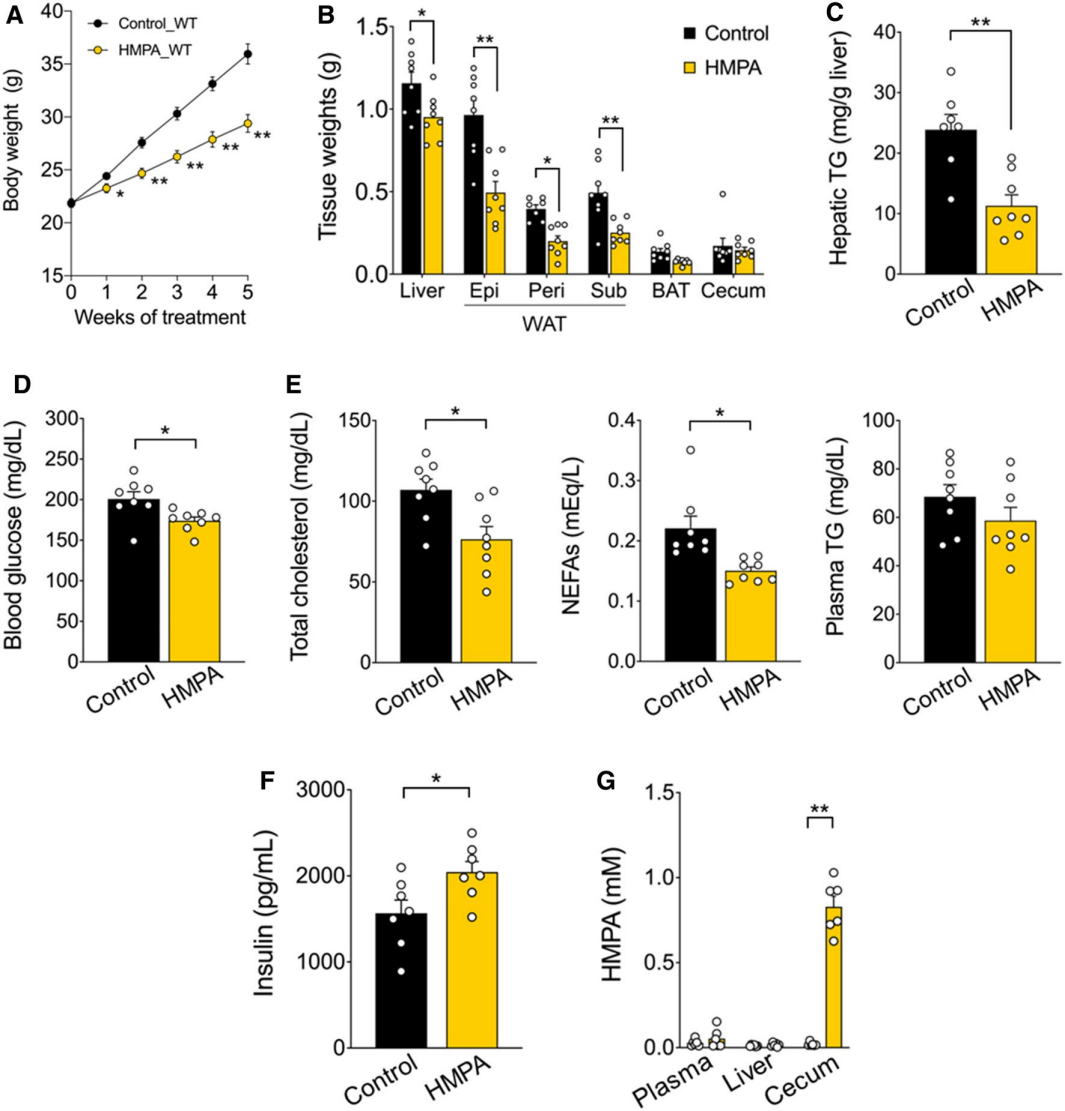
3-(4-Hydroxy-3-methoxyphenyl) propionic acid (HMPA) supplementation exerts metabolic benefits. (**A**) C57BL/6J wild-type male mice were fed HFD containing 1% HMPA for 5 weeks. Bodyweight gain during HMPA treatment (n  =  8 per group). (**B**) Mass of the liver, WAT, BAT, and cecum (n  =  8 per group). (**C**) Hepatic TG contents (control group, n  =  7; HMPA group, n  =  8). (**D**) Blood glucose level (n  =  8 per group). (**E**) Plasma levels of total cholesterol, NEFAs, and TG (n =  8 per group). (**F**) Plasma insulin level (n  =  7 per group). (**G**) HMPA levels in plasma, liver, and cecum samples after 5 weeks of HMPA-supplemented HFD feeding (n  =  6 per group). All data are presented as the mean ± standard error of the mean. **P < 0.01; *P < 0.05 (Mann–Whitney U test: **A**–**G**). HFD, high-fat diet; WAT, white adipose tissue; Epi, epididymal white adipose tissue; peri, perirenal white adipose tissue; sub, subcutaneous white adipose tissue; BAT, brown adipose tissue; TG, triglyceride; NEFAs, non-esterified fatty acids.

Initially, the effects of HMPA intake on the metabolic parameters in an HFD-induced obesity mouse model were examined. In this investigation, 7-week-old male mice were kept on HFD supplemented with 1% HMPA for 5 weeks (Table S1), and tissue properties and biochemical parameters were assessed after the administration period. Bodyweight gains were displayed as a substantial decrease in HMPA-supplemented HFD-fed mice during growth (Fig. 1A). The mass of the white adipose tissue (WAT) was also significantly lower in HMPA-supplemented HFD-fed mice than in control diet-fed mice; however, no significant difference was observed in the weight of the brown adipose tissue and the cecum (Fig. 1B). In addition, compared with control mice, HMPA-supplemented HFD-fed mice displayed a significant decrease in liver weight, which was accompanied by a reduction in hepatic triglyceride (TG) levels (Fig. 1B, C). Plasma levels of glucose, total cholesterol, and non-esterified fatty acids (NEFAs), but not TG levels, were significantly lower in HMPA-supplemented HFD-fed mice (Fig. 1D, E). Interestingly, at 12 weeks of age, plasma insulin levels were significantly higher in the HMPA-fed mice than those in control mice (Fig. 1F). HMPA intake resulted in the suppression of both an increase in plasma glucose levels and hepatic TG accumulation, effectively preventing HFD-induced obesity. However, after HMPA intake, high HMPA concentrations were detected in the cecum rather than in the plasma or liver (Fig. 1G), suggesting the need to investigate the absorption kinetics of HMPA when considering its effect on improving hepatic lipid metabolism.

### HMPA and lipid metabolism in the liver

**Figure 2:**
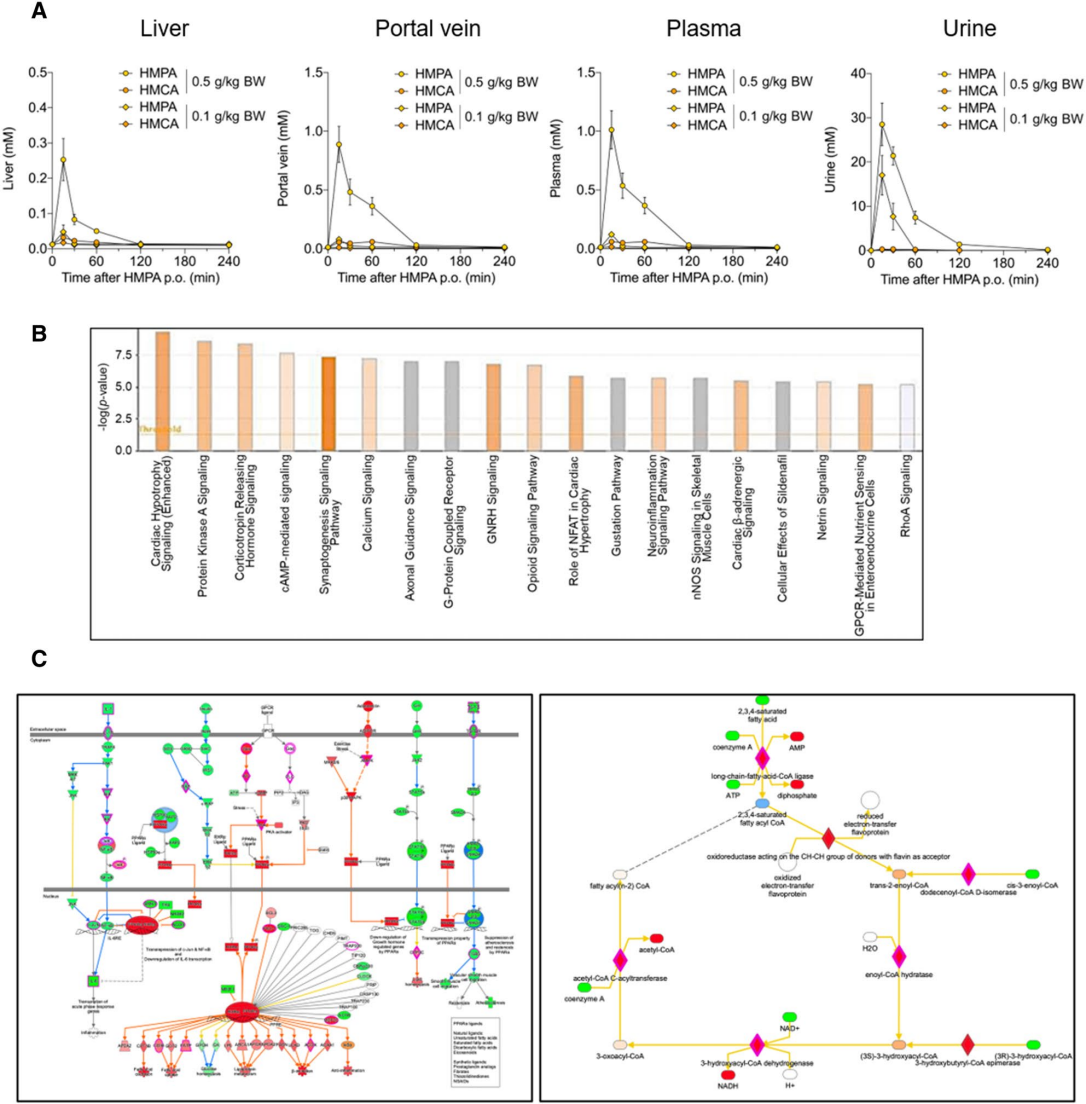
Pharmacokinetic profiles of HMPA and RNA-Seq transcriptome analysis of the liver following HMPA administration. (**A**) After oral administration of HMPA (0.1 and 0.5 g/kg bodyweight, respectively), HMPA and HMCA contents in the liver, portal vein, plasma, and urine samples were determined at 0, 15, 30, 60, 120, and 240 min after administration (n = 4). (**B**) Top canonical pathways by functional enrichment analysis of differentially expressed genes. P-values were adjusted based on the false discovery rate (FDR). (**C**) Gene networks including Upstream Regulator Analysis and Molecule Activity Predictor. Activation state of individual genes predicted by the Ingenuity Pathway Analysis with downstream predicted activation of protein kinase A (PKA) signaling. All data are presented as the mean ± standard error of mean. BW, bodyweight; p.o., per os.

Then, we identified and quantified HMPA levels among liver, portal vein, plasma, and urine samples collected at fixed time points between 0 and 240 min after the oral administration of HMPA. As shown in Fig. 2A, HMPA was successfully detected in all samples collected up to 15 min after its administration but not in samples collected at 240 min. As previously reported, orally administered HMPA was rapidly absorbed into the bloodstream and was mainly excreted in the urine after absorption into the body^24^. The determination of the area under the curve (AUC) from dosing until 240 min (AUC _0–240 min_) was calculated based on the HMPA amount accumulated at each time point. Following a dose of HMPA (0.5 g/kg body weight), the absorption amount in the body were 0.46 ± 0.08 nmol in the portal vein, 0.49 ± 0.12 nmol in plasma, and 0.08 ± 0.01 nmol in the liver. In addition, urinary excretion was 0.06 ± 0.01 nmol. Similarly, following oral administration of HMCA, it was also detected in the liver, portal vein, and plasma within the initial 15-min period (Fig. S1). After a single dose of HMCA (0.5 g/kg bodyweight), the absorption amount in the body were 0.25 ± 0.05 nmol in the portal vein, 0.26 ± 0.06 nmol in plasma, and 0.10 ± 0.02 nmol in the liver, with urinary excretion as 0.01 ± 0.001 nmol. Compared with HMCA, HMPA exhibited higher concentrations in the portal vein, plasma, and urine, implying a potentially higher absorption efficiency of HMPA (Figs. 2A and S1).

Therefore, RNA-sequencing (RNA-seq) analysis was performed to investigate the underlying mechanism through which HMPA administration enhances lipid metabolism in the liver. In total, eight RNA-seq libraries were constructed using RNA samples extracted from the livers of mice orally treated with HMPA or vehicle control mice. Thus, the differentially expressed genes (DEGs) defined as multiple platforms were analyzed with the Ingenuity pathway analysis (IPA) to identify statistically significant canonical pathways with a threshold of − Log (P-value) > 2, and canonical pathways were found to be enriched (Fig. 2B). The pathway of protein kinase A (PKA) signaling is among the top-ranked pathways, and this pathway is a cyclic AMP (cAMP)-dependent protein kinase involved in various physiological functions, including lipid metabolism^25^. In addition to canonical pathways, the regulatory effects analysis reveals potential pathways within upstream regulatory networks and downstream functions following HMPA administration using the IPA (Fig. S2). PKA activation in the liver following HMPA administration suggests the potential of promoting lipid breakdown through the downstream activation of the transcription factor PPARα, which is suggested to be a master transcriptional regulator of hepatic metabolic function (Fig. 2C).

### GPR41 as a specific receptor for HMPA

**Figure 3:**
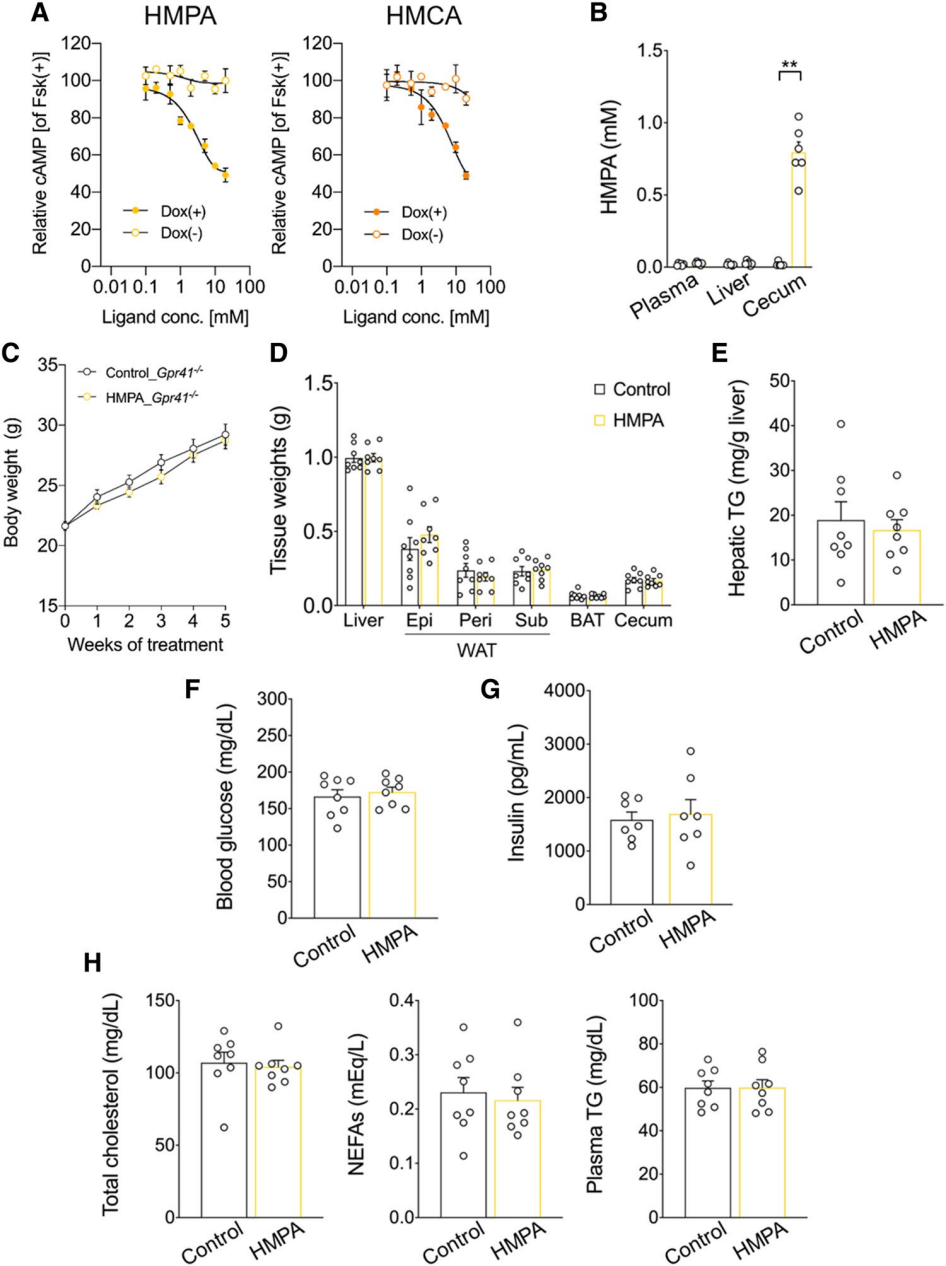
Affinity of HMPA for GPR41 and HMPA-mediated metabolic effects via GPR41. (**A**) cAMP inhibition assay for HMPA or HMCA using mouse-GPR41-expressing HEK293 cells. Cells were cultured for 24 h and then treated with or without 10 μg/mL of doxycycline (Dox) (n = 6 independent cultures). All data are presented as relative to forskolin (Fsk)-induced cAMP levels. Closed symbols represent values from cells treated with Dox, and open symbols denote untreated groups. (**B**) *Gpr41*^*−/−*^ male mice were fed HFD containing 1% HMPA for 5 weeks. HMPA contents among plasma, liver, and cecum (n = 6 per group). (**C**) Bodyweight gain during HMPA treatment (n = 8 per group). (**D**) Mass of the liver, WAT, BAT, and cecum (n = 8 per group). (**E**) Hepatic TG contents (n = 8 per group). (**F**) Blood glucose level (n = 8 per group). (**G**) Plasma insulin level (n = 7 per group). (**H**) Plasma levels of total cholesterol, NEFAs, and TG (n = 8 per group). All data are presented as the mean ± standard error of the mean. **P < 0.01; *P < 0.05 (Mann–Whitney U test). HFD, high-fat diet; WAT, white adipose tissue; Epi, epididymal white adipose tissue; peri, perirenal white adipose tissue; sub, subcutaneous white adipose tissue; BAT, brown adipose tissue; TG, triglyceride; NEFAs, non-esterified fatty acids.

HMPA absorbed into the body may potentially suppress TG accumulation by activating the signaling of hepatic lipid degradation pathways. To identify such host-sensing and signaling mechanisms, host receptors responsible for recognizing HMPA were explored. Considering the chemical structure of HMPA (Fig. S3A), which belongs to the phenylpropanoid family and contains a propionic acid moiety attached to the phenyl ring, HMPA may have the ability to interact with SCFA receptors, such as propionic acid. To test this hypothesis, the ligand affinity of HMPA for GPR41 and GPR43 receptors was evaluated. In human embryonic kidney 293 (HEK293) cells expressing mouse GPR41, HMPA significantly suppressed cAMP levels induced by forskolin in a dose-dependent manner (Fig. 3A), whereas such activation was not found in GPR43 (Fig. S3B) or not observed in doxycycline-untreated controls [Dox (–)]. On the contrary, HMCA also selectively exhibits affinity for GPR41, with an EC_50_ of 5.58 mM, although HMPA demonstrated an even higher affinity than HMCA, with an EC_50_ of 2.36 mM (Figs. 3A and S3B). Collectively, these results suggest that HMPA has the potential to function as a selective GPR41 agonist.

### HMPA-mediated metabolic effects via GPR41

Then, we investigated whether HMPA exerts anti-obesity effects and improves hepatic lipid metabolism via GPR41 in HFD-induced obesity model using *Gpr41*-deficient male mice (*Gpr41*^*−/−*^ mice). GPR41 is activated by SCFAs such as propionate produced by the gut microbial fermentation of dietary fiber and contributed to the regulation of energy homeostasis^21,26^. Although HMPA concentrations were high in the cecum of *Gpr41*^*−/−*^ mice after a 5-week intake of HFD containing 1% HMPA (Fig. 3B), *Gpr41*^*−/−*^ mice failed to benefit from the HMPA treatment, as shown by the HFD-induced weight gain and WAT mass increase compared with the control group (Fig. 3C, D). Concomitantly, the absence of alterations in hepatic steatosis suggested GPR41’s involvement in the regulation of HMPA-mediated hepatic lipid metabolism (Fig. 3E). Finally, HMPA-treated *Gpr41*^*−/−*^ mice had comparable plasma levels of glucose and insulin (Fig. 3F, G), and plasma parameters of lipid metabolism were unchanged when compared with those in control mice (Fig. 3H).

### HMPA as a molecular entity underlying metabolic improvement

**Figure 4:**
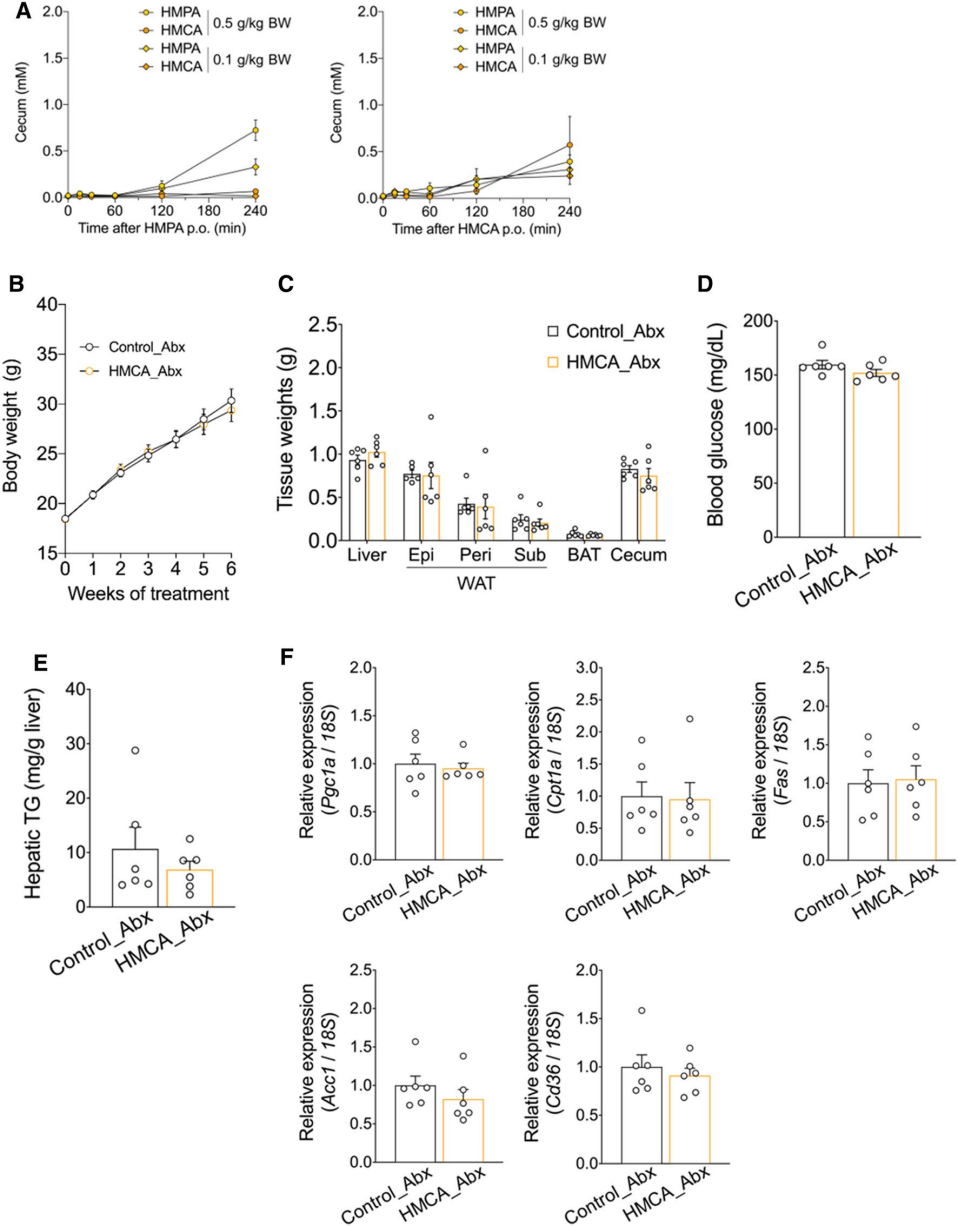
Metabolic parameter changes in HMCA-supplemented HFD-fed mice during antibiotic treatment. (**A**) After the oral administration of HMPA (left) or HMCA (right) (0.1 and 0.5 g/kg bodyweight, respectively), HMPA and HMCA contents in the cecum were determined (n  =  4). (**B**) For 5 weeks, antibiotics-treated C57BL/6J wild-type male mice were fed HFD containing 1% HMCA. Bodyweight gain during HMCA treatment (n  =  6 per group). (**C**) Mass of the liver, WAT, BAT, and cecum (n  =  6 per group). (**D**) Blood glucose level (n  =  6 per group). (**E**) Hepatic TG contents (n =  6 per group). (**F**) Relative mRNA expressions involved in lipid metabolism in the liver of HFD control mice and HMCA-supplemented HFD-fed mice (n  =  6 per group). The results are presented as the fold change in mRNA expression relative to the HFD control, with a value of 1 assigned to the control group. To compare the mRNA expression levels across different samples, the copy numbers of all transcripts were normalized to the expression levels of *18S* mRNA as an internal control. All data are presented as the mean ± standard error of mean. BW, bodyweight; HFD, high-fat diet; WAT, white adipose tissue; Epi, epididymal white adipose tissue; peri, perirenal white adipose tissue; sub, subcutaneous white adipose tissue; BAT, brown adipose tissue; TG, triglyceride; Abx, antibiotics.

Previously, we showed that dietary HMCA was efficacious against HFD-induced weight gain and hepatic steatosis, and the gut microbiota convert HMCA into HMPA in the host intestine^11^. To investigate whether these metabolic benefits of HMCA were responsible for gut microbiota-produced HMPA, a comprehensive analysis of various metabolic phenotypes was performed in a model of antibiotic-treated male mice fed HFD with HMCA supplementation. The results confirmed the metabolism and conversion of HMPA from HMCA in the intestine. After oral administration of two doses of HMCA in mice, not only HMCA but also HMPA were detected at 240 min in the cecum concentration–time curves (Fig. 4A). By contrast, when HMPA was administered orally, only HMPA was detected in the cecum (Fig. 4A). These results indicate that HMPA is produced in the intestine after HMCA intake, and HMPA exhibited a biphasic absorption profile in the portal vein and liver (Fig. S4A).

Expectedly, HMPA was detected at extremely low concentrations not only in the plasma but also in the liver and cecum of antibiotic-treated mice after HMCA intake (Fig. S4B). In accordance with these results, the anti-obesity effect of HMCA was abolished when administered to antibiotic-treated mice (Fig. 4B). Furthermore, no significant differences were observed in the weights of the WAT and liver, and plasma glucose levels were unchanged when compared between the groups (Fig. 4C, D). Under antibiotic therapy, HMCA intake did not result in any changes in hepatic TG accumulation, and no changes were found in hepatic lipid metabolism-related genes such as *Pgc1a*, *Cpt1a*, *Fas*, *Acc1*, and *Cd36* (Fig. 4E, F). Thus, the conversion of HMCA into HMPA by gut microbiota indicated the potential importance of HMPA as a key molecule that contributes to the anti-obesity effect and hepatic lipid metabolism.

## Discussion

Growing evidence shows that the gut microbiota transforms and modify dietary phenolic compounds, and their microbial metabolites exert beneficial effects on host metabolic homeostasis^3, 5^. However, the specific HMPA receptors and functions have not been identified to unravel their pathophysiological implications. This study used mouse models of HFD-induced obesity and confirmed the identification and unraveling of GPR41 for gut microbiota-produced HMPA from HMCA, which contributed to the anti-obesity effects and improved hepatic steatosis through evoked lipid catabolism pathway following the activation of the PKA–PPARα cascade. Furthermore, our analysis of the bioaccessibility and bioavailability dynamics of HMPA showed that HMPA has higher absorption efficiency than HMCA and exhibits a higher affinity for GPR41. These findings present that HMPA, as a gut microbial metabolite, may be one of the molecular entities underlying the anti-obesity and metabolic improvement effects observed with dietary phenolic compounds.

In this study, we have demonstrated the potential of HMPA to undergo metabolic conversion from HMCA by gut microbiota using antibiotic-treated mice and have elucidated its absorption and distribution profile. Based on the estimations of absorption^27^ and total plasma volume in the animal (equivalent to 4.5% of body weight)^28^, the calculated absorption ratio of HMPA and HMCA in plasma was 0.84 ± 0.13% and 0.43 ± 0.04%, respectively, following oral administration at a dose of 0.5 g/kg body weight (Figs. 2A and S1). Our analysis showed that HMPA had a higher absorption rate than HMCA. Several investigations have shown that HMPA is effectively transported through the monocarboxylic acid transporter in the intestinal tract^29^. Additionally, the pharmacokinetic analysis suggested that absorbed HMPA might rapidly (Tmax, 15 min) be excreted through urine. According to Abe et al.^24^, absorbed HMPA and derived glucuronide and sulfate conjugated forms were detected at the highest concentration in the kidney rather than in the liver 15 min after intake, indicating predominant renal excretion over hepatic metabolism. However, the presence of HMPA in the cecum after 5 weeks of exposure to HMPA suggests the role of unabsorbed HMPA-related metabolites from the gut microbiota in regulating metabolic function. In human studies, after coffee intake, human blood and urine samples had HMPA and related metabolites, such as dihydrocaffeic acid and hydroxyhippuric acid, which are recognized as gut microbial metabolites, in concentrations spanning from micromolar to millimolar levels^30^. Consequently, HMPA and its related metabolites may play a significant role in promoting human health. By contrast, HMCA absorbed from the intestine is transformed into various derivatives such as their -glucuronide and -sulfate conjugated forms and HMPA in the liver^24,29^. Furthermore, HMCA can be metabolized into several other forms, such as isoferulic acid (IFA) in the colon^31^. Considering the reported physiological effects of these HMCA-derived metabolites, a comprehensive evaluation of these metabolites and HMPA is needed.

For the first time, we have demonstrated the potential of GPR41 as a receptor for polyphenolic molecules. GPR41 and GPR43 are activated by SCFAs such as acetic acid, propionic acid, and butyric acid, which are produced by gut microbiota from dietary fibers in the colon. The EC_50_ for GPR41 was higher with HMPA compared with that with SCFA (EC_50_ of GPR41 with HMPA, 2.36 mM; with SCFA, 6–127 μM^20^). However, a single-dose trial of HMPA revealed its potential absorption at millimolar levels in the bloodstream. Additionally, the observed anti-obesity and hepatic lipid metabolism improvement effects following HMPA administration were evident only in wild-type mice, not in *Gpr41*^*−/−*^ mice, indicating that HMPA acts as a molecular entity through GPR41. Previous studies have reported that GPR41 exhibits the highest affinity for propionic acid, whereas GPR43 has the highest affinity for acetic acid^20^. Therefore, HMPA and HMCA, defined by phenylpropanoid, may selectively exhibit affinity for GPR41 but not GPR43. HMPA, as a reduced form of HMCA, lacks the unsaturated bond in the propionyl group of the phenyl ring, presenting that HMPA may exhibit higher agonistic activity for GPR41 than HMCA. Despite HMCA being a ligand for GPR41, no anti-obesity or improvement in hepatic lipid metabolism was observed when HMCA was administered to antibiotic-treated mice without HMPA production. These findings could be due to the limited bioavailability of HMCA, resulting in insufficient concentrations necessary to activate GPR41 in the absence of HMPA production in antibiotic-treated mice. In the liver, HMCA absorbed from the intestine is converted into various derivatives other than HMPA^32^. Therefore, whether other HMCA-derived metabolites such as IFA can also act as ligands for GPR41 must be also evaluated, providing valuable insights into the mechanisms of metabolic improvement effects by phenolic compounds.

Our results show HMPA may contribute to the improvement of hepatic lipid metabolism through GPR41 in an HFD-induced obesity model using *Gpr41*^*−/−*^ mice. GPR41 is coupled with the Gi/o protein, which decreases the intracellular concentration of cAMP as demonstrated by the cAMP inhibition assay for HMPA using mouse-GPR41-expressing HEK293 cells (Fig. 3A). GPR41 is highly expressed in sympathetic ganglia located in the portal vein, and its SCFA-induced stimulation promotes the release of norepinephrine from sympathetic neurons, contributing to the regulation of energy metabolism^21^. By contrast, RNA-seq analysis for the liver has revealed the activation of Gαs signaling, which is upstream of the PKA pathway, following HMPA administration (Fig. 2B, C). Thus, HMPA may not directly target GPR41 in the liver but exert its effects on GPR41 located in the sympathetic ganglia within the portal vein. Consequently, Gαs activation is a potential outcome of the indirect influence of HMPA on the liver through the sympathetic efferent pathway mediated by the central nervous system. Although the HMPA-mediated regulation of hepatic lipid metabolism implies the involvement of inter-organ communication mechanisms, further experiments are needed to determine tissue-specific effects of HMPA on the abrogation of hepatic steatosis.

In this study, HMPA-stimulated GPR41 activation exhibits superiority over HMCA in contributing to the anti-obesity effects and suppression of hepatic steatosis. Furthermore, RNA-seq analysis unveiled the activation of lipid metabolism-related genes in the liver following HMPA administration, concurrently verifying the potential of HMPA-mediated GPR41-signaling to affect the liver via inter-organ communication. By using tissue-specific *Gpr41*^*−/−*^ mice, the identification of the target organs directly affected by HMPA through GPR41 warrants detailed follow-up investigations and further characterization of a sensitive and exhaustive analysis of various metabolites in addition to HMPA after HMCA administration. Our findings may have significant contributions to the development of functional food targeting the prevention of metabolic disorders, including obesity, type 2 diabetes mellitus, and non-alcoholic steatohepatitis. Furthermore, our study provides valuable insights into the promotion of preventive pharmaceuticals targeting for GPR41 against metabolic disorders.

## Methods

### Animal study

Male C57BL/6J wild-type mice (Japan SLC, Shizuoka, Japan) were housed in a conventional animal room at 23.0 °C with a 12-h light–dark cycle. The generation of *Gpr41*^*−/−*^ mice was described previously^21^. Before starting the experiments, the mice were acclimated to the laboratory conditions and fed the CLEA Rodent Diet (CE-2, CLEA Japan, Tokyo, Japan). Then, 7-week-old male mice were divided into two groups of comparable average bodyweight [HFD group (D12492, 60% kcal fat; Research Diets, Inc., NJ, USA) supplemented with 1% cellulose (FUJIFILM Wako, Osaka, Japan) or 1% HMPA (Tokyo Chemical Industry, Tokyo, Japan)] for 5 weeks. After feeding, plasma, liver tissue, and cecal levels of HMPA were determined. For the antibiotic treatment, 7-week-old male mice were treated with 0.4 mg/mL of ampicillin (FUJIFILM Wako), 0.4 mg/mL of neomycin (Sigma-Aldrich, St. Louis, MO, USA), 0.4 mg/mL of metronidazole (FUJIFILM Wako), and 0.2 mg/mL of vancomycin (FUJIFILM Wako) in drinking water during HFD feeding. Mice treated with antibiotics were fed HFD containing 1% cellulose or 1% HMCA (FUJIFILM Wako) for 5 weeks. During the experiments, bodyweights were measured once a week. For the pharmacokinetic profiles of HMPA, 7-week-old male mice were orally administered with individual HMPA or HMCA (0.1 and 0.5 g/kg bodyweight, respectively), and HMPA contents in the liver, portal vein, plasma, urine, and cecum were sequentially evaluated. Blood was collected from the inferior vena cava using heparin-treated tubes, and the plasma was separated by immediate centrifugation (7000 *g*, 5 min, 4 °C). The compositions of the diets are provided in Table S1.

### Plasma biochemical analyses

Blood glucose concentrations were measured using a One Touch Ultra (LifeScan). The plasma levels of total cholesterol, NEFAs, and TG were analyzed using commercial kits (total cholesterol, LabAssay Cholesterol; free fatty acids, LabAssay NEFA; and TG, LabAssay Triglyceride; FUJIFILM Wako). Insulin levels were measured using commercial kits [ELISA Kit (FUJIFILM Wako)], following the manufacturer’s instructions.

### Cell culture

The generation of GPR41-expressing HEK293 cells was described previously^21^, and cells were cultured in Dulbecco’s modified eagle’s medium supplemented with 10 μg/mL of blasticidin S (Funakoshi), 100 μg/mL of hygromycin B (Thermo Fisher Scientific, MA, USA), and 10% fetal bovine serum at 37°C with 5% CO_2_. For cyclic AMP (cAMP) determination, Flp-In T-Rex HEK 293 transfected with a mixture containing mouse GPR41 or GPR43 cDNA in the pcDNA5/FRT/TO vector and the pOG44 vector were plated in 24-well plates at a density of 1 × 10^5^ cells per well. After 24 h of culture, the cells were treated with or without doxycycline (10 μg/mL, Sigma-Aldrich) for an additional 24 h. To upregulate the cAMP levels, the cells were treated with 2 μM forskolin (Sigma-Aldrich) and 500 μM of 3-isobutyl 1-methylxanthine (Sigma-Aldrich). They were then stimulated with each concentration of HMPA or HMCA (0.1, 0.2, 0.5, 1.0, 2.0, 5.0, 10, and 20 mM) for 10 min. The cAMP levels were determined using the cAMP EIA kit (Cayman Chemical, MI, USA), following the manufacturer’s instructions.

### Quantification of phytochemicals by high-performance liquid chromatography

HMPA and HMCA levels in the plasma, portal vein, and urine, liver, and cecum samples were measured following a previously described protocol with modifications^11^. Mouse plasma and tissue samples were dissolved or homogenized in an acetonitrile-containing extraction solvent. The samples were centrifuged at 13,000 *g* and 4 ℃ for 5 min. The supernatants containing HMPA and HMCA were collected and subjected to liquid chromatography (LC) with tandem mass spectrometry using an ultra-performance LC system (UPLC, Waters Corporation, MA, USA) equipped with an Acquity UPLC system coupled to a Waters Xevo TQD mass spectrometer (Waters Corporation). Injections were performed with an autosampler maintained at 4 ℃. The mobile phase was pumped at a flow rate of 0.3 mL/min. The samples were separated on an ACQUITY UPLC BEH C18 column (2.1 × 150 mm, 1.7 μm; Waters Corporation) using a methanol gradient in 0.1% formic acid aqueous solution.

### RNA isolation and quantitative reverse transcriptase polymerase chain reaction (qRT-PCR)

Total RNA was isolated using the RNAiso Plus reagent (Takara Bio, Shiga, Japan). Complementary DNA (cDNA) synthesis was carried out using the RNA as a template and Moloney murine leukemia virus reverse transcriptase (Invitrogen, MA, USA). For qRT-PCR analysis, SYBR Premix Ex Taq II (Takara Bio) and the StepOne real-time PCR system (Applied Biosystems, MA) were employed, following previously described protocols^21,22^. The PCR protocol consisted of an initial denaturation step at 95 °C for 30 s, followed by 40 cycles of amplification comprising denaturation at 95 °C for 5 s, annealing at 58 °C for 30 s, and extension at 72 °C for 1 min. Each sample was tested twice, and the average cycle threshold (Ct) value was determined. To calculate the relative mRNA expression, normalization to the *18S* rRNA reference gene was performed using the 2^(−ΔΔCt)^ method. The primer sequences used for amplifying the target genes were as follows: *18S*, Forward: 5′-CTCAACACGGGAAACCTCAC-3′, Reverse: 5′-AGACAAATCGCTCCACCAAC-3′; *Fas*, Forward: 5′-GCTGCGGAAACTTCAGGAAAT-3′, Reverse: 5′-AGAGACGTGTCACTCCTGGACTT-3′; *Acc1,* Forward: 5′-AAGGCTATGTGAAGGATG-3′, Reverse: 5′-CTGTCTGAAGAGGTTAGG-3′; *Cd36*, Forward: 5′-TGGCAAAGAACAGCAGCAAA-3′, Reverse: 5′-GACAGTGAAGGCTCAAAGATGG-3′; *Cpt1a*, Forward: 5′-GCATAAACGCAGAGCATTCC-3′, Reverse: 5′-GATGTTGGGGTTCTTGTCTCC-3′; *Pgc1a*, Forward: 5′-GAGAATGAGGCAAACTTGCTAGCG-3′, Reverse: 5′-TGCATGGTTCTGAGTGCTAAGACC-3′.

### RNA-seq

RNA was extracted from the liver of HMPA (2.0 g/kg bodyweight)-treated mice using an RNAiso Plus reagent (Takara Bio) and RNeasy mini kit (Qiagen, Germany). RNA-seq libraries were generated with the TruSeq Stranded mRNA Library Prep Kit (Illumina, CA, USA) and sequenced on NovaSeq 6000. Approximately 4-Gb paired-end reads of 100-bp length per sample were obtained. RNA-seq data were preprocessed using Trimmomatic to remove adapters or poor-quality reads, and trimmed sequences were assessed using FastQC^33,34^. The reads were aligned to the Illumina iGenomes NCBI GRCm38. To obtain DEGs from all comparisons, raw read counts were subjected to relative log expression normalization. Data expressed as fold change of DEGs were identified based on the following criteria: false discovery rate (FDR)-adjusted P-value < 0.05 (using the Benjamini–Hochberg procedure) and log2 (fold change) > 0.5. A gene set enrichment analysis was performed using Bioconductor version 3.0. The Gene Ontology terms of molecular function, biological process, cellular component, and pathway were considered. IPA (http://ingenuity.com/index.html) was used to identify canonical pathways and gene networks including Upstream Regulator Analysis and Molecule Activity Predictor.

### Statistical analysis

All data are presented as the mean ± standard error of the mean. The normality of the data was assessed using the Shapiro–Wilk test. For comparisons between two groups, the Student t-test or the Mann–Whitney U test was applied to determine statistical significance. When comparing multiple groups (≥ 3 groups), the one-way analysis of variance followed by Dunnett’s test or the Kruskal–Wallis test followed by Dunn’s post-hoc test was used. Statistical differences were considered significant at a P-value of less than 0.05. The FDRs of the RNA-seq data were estimated using the Benjamini–Hochberg procedure.

### Ethics statement

All mouse-associated procedures were conducted in accordance with the approved protocols of the Kyoto University Animal Experimentation Committee (Lif-K22015) and the Tokyo University of Agriculture and Technology (Permit no. 28-87) for ethical treatment of animals. All methods were approved by the Kyoto University Animal Experimentation Committee (Lif-K22015) and the Tokyo University of Agriculture and Technology (Permit no. 28-87). Mice were euthanized under deep anesthesia using isoflurane, and all efforts were made to minimize any potential discomfort or suffering experienced by the animals. All experiments were performed in accordance with ARRIVE guidelines (https://arriveguidelines.org). All methods were performed in accordance with the relevant guidelines and regulations.

### Supplementary Information

Supplementary Information.

## Data Availability

The source data presented in RNA-seq analysis have been deposited into the Dryad repository (https://doi.org/doi:10.5061/dryad.z612jm6hd.). All other data that support the findings of this study are available from the corresponding author upon reasonable request.
