# Supplementary material for: 3-(4-Hydroxy-3-methoxyphenyl) propionic acid contributes to improved hepatic lipid metabolism via GPR41
Source: Sci Rep. 2023 Dec 1;13:21246. doi: 10.1038/s41598-023-48525-3 (PMC10692101; doi:10.1038/s41598-023-48525-3)
Supplement: Supplementary file 1 — Supplementary Information. [file 41598_2023_48525_MOESM1_ESM.pdf]

SUPPLEMENTARY INFORMATION

3-(4-Hydroxy-3-methoxyphenyl) propionic acid contributes to improved hepatic lipid metabolism via GPR41

Ryuji Ohue-Kitano, Yuki Masujima, Shota Nishikawa, Masayo Iwasa, Yosuke Nishitani, Hideaki Kawakami, Hiroshige Kuwahara, and Ikuo Kimura

Corresponding author:
Ryuji Ohue-Kitano
Ikuo Kimura

This file includes:
Figures S1–4
Table S1

Supplemental Figure 1

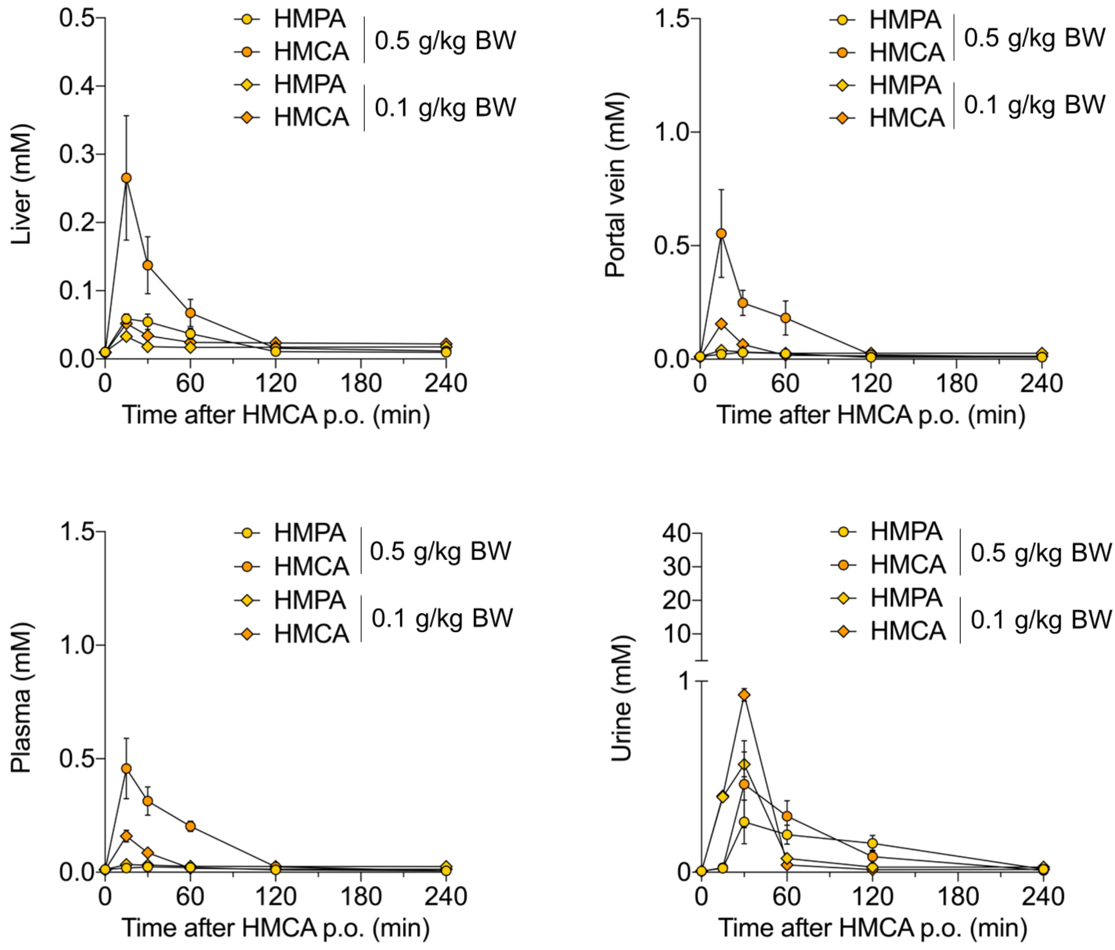

Supplemental Figure 1. Pharmacokinetic profiles following oral administration of HMCA. After oral administration of HMCA (0.1 and 0.5 g/kg bodyweight, respectively), HMPA and HMCA contents among liver, portal vein, plasma, and urine were determined at 0, 15, 30, 60, 120, 240 min post-administration (n = 4). All data are presented as the mean ± standard error of mean. BW, bodyweight; p.o., per os.

Supplemental Figure 2

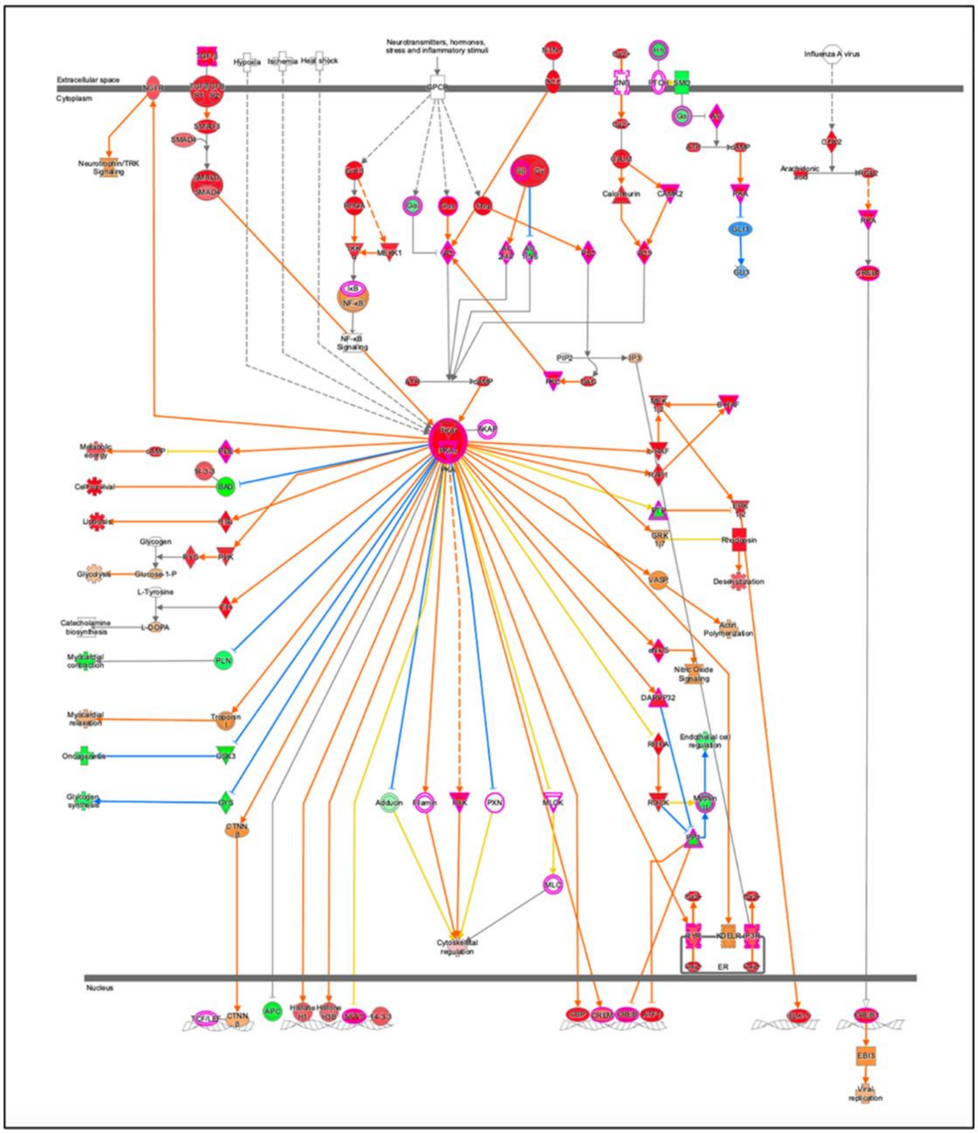

Supplemental Figure 2. Functional enrichment analysis of differentially expressed genes in the liver of mice orally treated with HMPA or vehicle control mice. Gene networks including Upstream Regulator Analysis and Molecule Activity Predictor for “Protein kinase A signaling".

Supplemental Figure 3

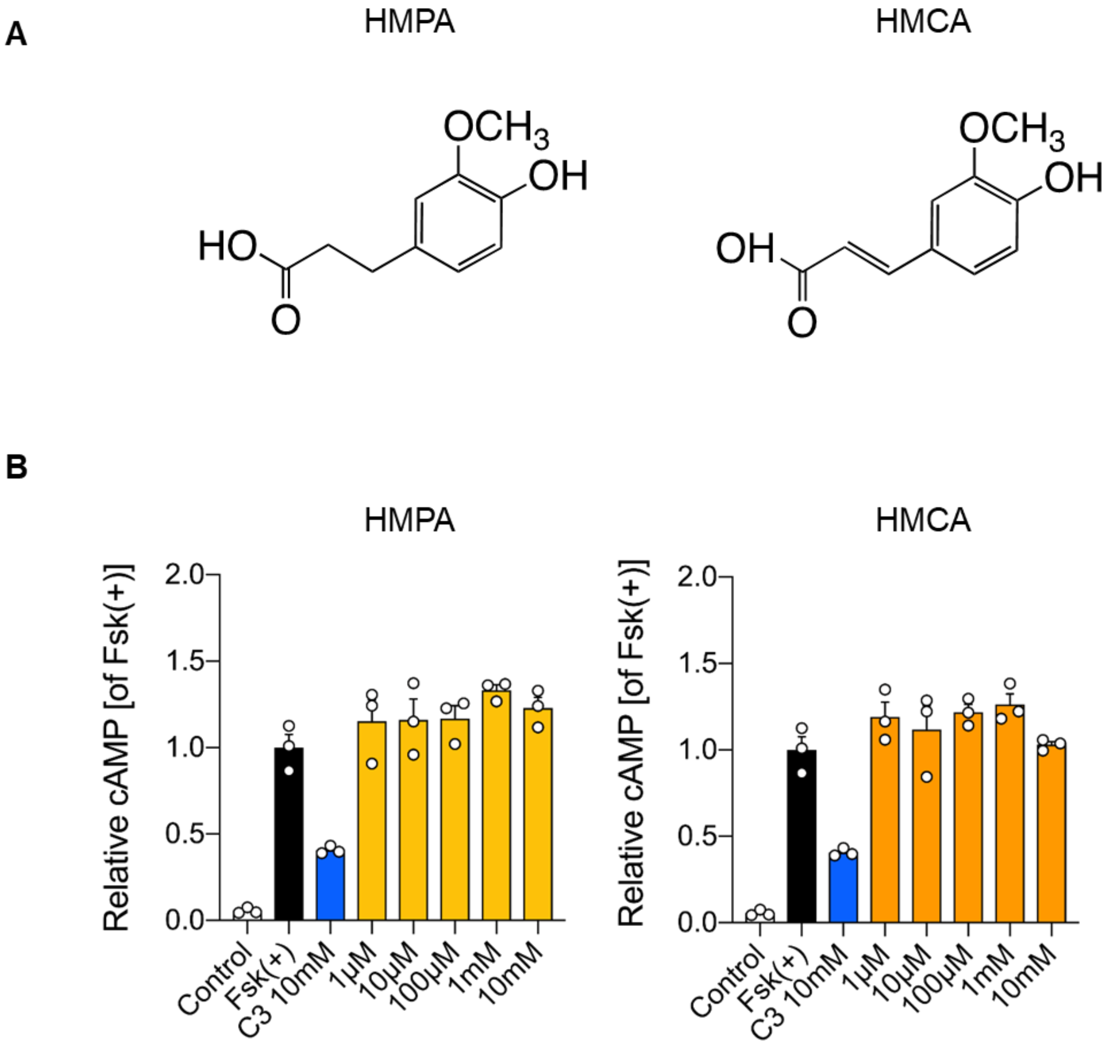

Supplemental Figure 3. Affinity of phytochemicals for GPR43. (**A**) Chemical structures of HMPA and HMCA. (**B**) cAMP inhibition assay for HMPA (left) or HMCA (right) using mouse-GPR43-expressing HEK293 cells. Cells were cultured for 24 h, and then treated with 10 μg/mL of doxycycline (n = 3 independent cultures). All data are presented as relative to forskolin (Fsk)-induced cAMP levels. Propionic acid (C3) is used as the positive control for GPR43 agonist. All data are presented as the mean ± standard error of mean.

Supplemental Figure 4

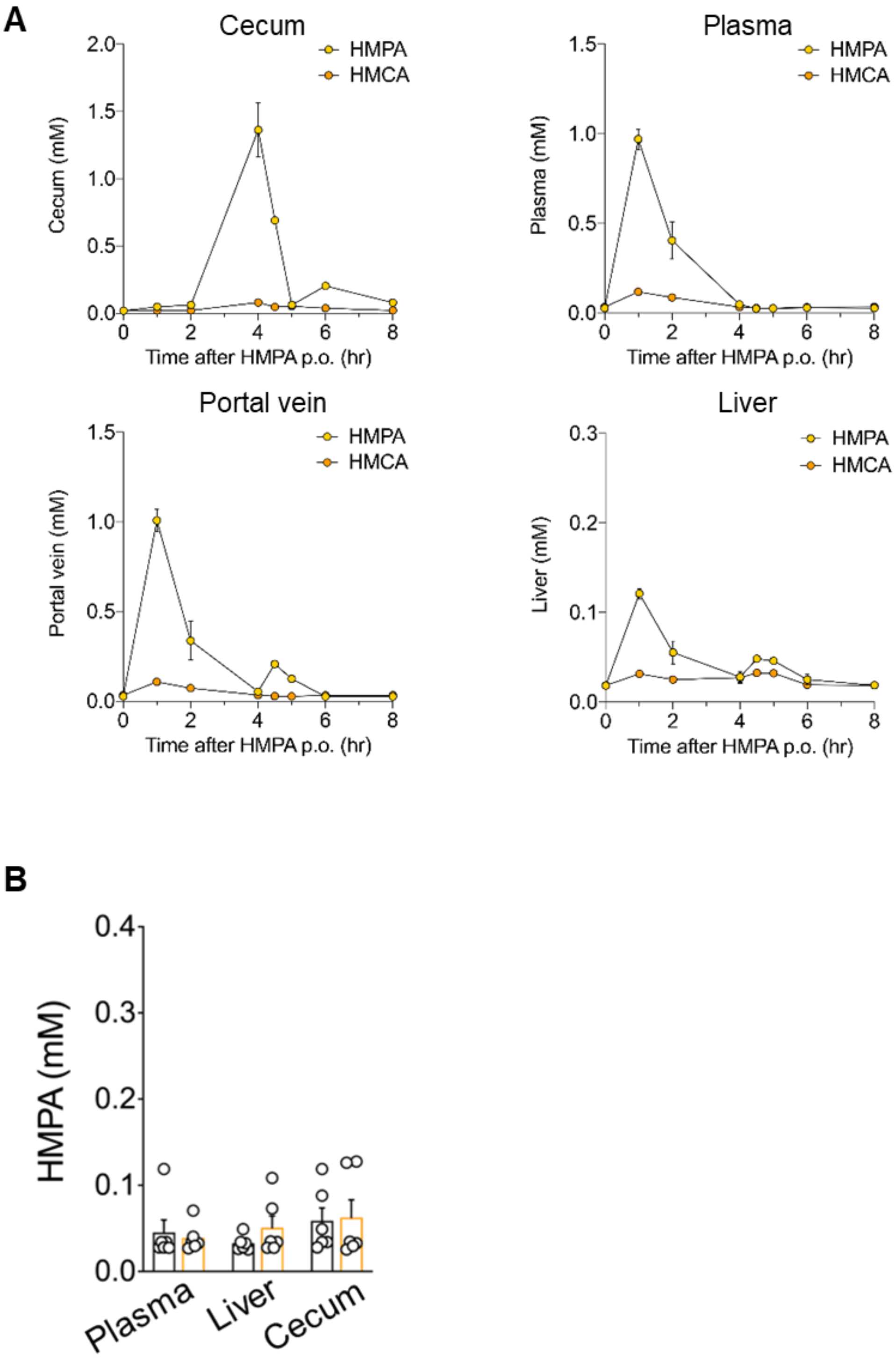

Supplemental Figure 4. Pharmacokinetic profiles of HMPA. (**A**) After oral administration of HMPA (1.0 g/kg bodyweight, respectively), HMPA or HMCA contents among cecum, plasma, portal vein, and liver were determined at 0, 1, 2, 4, 4.5, 5, 6, and 8-hour post-administration (n = 4). (**B**) HMPA contents among plasma

liver, and cecum after 5-week HMCA-supplemented HFD-feeding during antibiotic treatment (n = 6 per group). All data are presented as the mean ± standard error of mean. p.o., per os.

Supplemental Table 1 Diet compositions.

Formula	D12492	Control	HMPA	HMCA
Product	gm%	gm%	gm%	gm%
Protein	20	20	20	20
Carbohydrate	20	20	20	20
Fat	60	60	60	60
Ingredient	gm	gm	gm	gm
Casein, 30 Mesh	200	198	198	198
L-Cystine	3	2.97	2.97	2.97
Corn Starch	0	0	0	0
Maltodextrin 10	125	123.75	123.75	123.75
Sucrose	68.8	68.112	68.112	68.112
Cellulose, BW200	50	49.5	49.5	49.5
Cellulose	0	7.7385	0	0
HMPA	0	0	7.7385	0
HMCA	0	0	0	7.7385
Soybean Oil	25	24.75	24.75	24.75
Lard	245	242.55	242.55	242.55
Mineral Mix S10026	10	9.9	9.9	9.9
DiCalcium Phosphate	13	12.87	12.87	12.87
Calcium Carbonate	5.5	5.445	5.445	5.445
Potassium Citrate, 1 H2O	16.5	16.335	16.335	16.335
Vitamin Mix V10001	10	9.9	9.9	9.9
Choline Bitartrate	2	1.98	1.98	1.98
FD&C Blue Dye #1	0.05	0.0495	0.0495	0.0495
